# Temperature of Rooms

**Published:** 1866-12

**Authors:** 


### TEMPERATURE OF ROOMS.

Ordinarily we are comfortable in church, if at the height of five feet from the floor, in the centre of the building, Fahrenheit's thermometer stands at sixty-five degrees. But in this respect no one man should be a guide for another. Some require more heat than others, but there is one rule of universal application—a rule which admits of no exceptions, the world over, each person should notice what temperature keeps him comfortably warm, and thus be a rule to himself. It is said of the Duke of Wellington that during the latter years of his life he became so frigid, that in order to be comfortably warm his room was kept at such a temperature in winter that it was in a measure impossible for any visitor to remain but a very few minutes.

But when a man has taken a cold, or is becoming bilious; or if he stays indoors several days, he requires more and more heat, and if under such circumstances he would eat positively nothing for a day or two and keep on piling up the wood so as to keep up a continual slight perspiration, the cold would be cut short off, or the biliousness would disappear in twenty-four hours; in fact, very many of our aches and pains and ailments would disappear with an amazing promptness, if we could persuade ourselves, when they are first noticed, to only cease eating, keep warm, keep quiet, and drink abundantly of any hot liquid; but the great misfortune is that nine persons out of ten prefer to take some kind of medicine however nauseous; they feel as if they could not spare the time to be sick, and would rather swallow a quart of the most disgusting compound, if it only promises to cure them up "right away," with the result always, that they are not cured right away; but after dosing themselves for days and weeks with whatever Tom, Dick or Harry chooses to advise, they find themselves compelled at last to consult a physician when the time has passed for warmth and quiet to have any curative effect.

